# Systems Bioengineering of Septic Shock Metabolism: Citrulline, β-Hydroxybutyrate and Plasma Biomarker-Based Phenotyping

**DOI:** 10.3390/biom16081189

**Published:** 2026-08-14

**Authors:** Leonard Azamfirei, Vlad Dimitrie Cehan, Alina Roxana Cehan, Mihai Claudiu Pui, Alexandra Lazar

**Affiliations:** 1 Anesthesiology and Intensive Care Department, George Emil Palade University of Medicine, Pharmacy, Science and Technology of Targu Mures, 540142 Targu Mures, Romania; 2 Doctoral School of Medicine and Pharmacy, George Emil Palade University of Medicine, Pharmacy, Science and Technology of Targu Mures, 540142 Targu Mures, Romania; 3 Plastic and Reconstructive Surgery, Emergency Clinical County Hospital of Targu Mures, 540136 Targu Mures, Romania; 4 Department of Anesthesiology and Intensive Care, County Emergency Clinical Hospital of Targu Mures, 540136 Targu Mures, Romania

**Keywords:** septic shock, systems bioengineering, artificial intelligence, citrulline, β-hydroxybutyrate, plasma biomarkers, metabolic phenotyping, metabolomics, mitochondrial dysfunction, precision medicine

## Abstract

Background: Although advances in critical care have improved short-term outcomes, sepsis survivors continue to face substantial chronic morbidity and impaired long-term survival. Conventional threshold-based tools such as the Sequential Organ Failure Assessment (SOFA) and Modified Early Warning Score (MEWS) show moderate and variable discrimination across cohorts. Reported areas under the receiver operating characteristic curve (AUROCs) must therefore be interpreted in relation to the population, prediction horizon, and outcome used in each study rather than as direct head-to-head comparisons. Objectives: This review evaluates how artificial intelligence (AI) could be linked with dynamic plasma metabolites, particularly citrulline and β-hydroxybutyrate (3-HB), to support biologically informed sepsis phenotyping, while critically examining mechanistic evidence, clinical limitations, and translational readiness. Data Synthesis: Machine-learning and natural language processing architectures have shown promising discrimination in many early-detection studies, with pooled AUROCs near 0.87 and reported prediction windows extending to 48 h. However, performance estimates vary with cohort composition, outcome definition, and validation design, and they should not be ranked against unrelated biomarker studies. Human sepsis studies generally associate low or persistently low citrulline with impaired intestinal function and organ injury, but no sepsis-specific decision cutoff has been externally validated. For 3-HB, an AUROC of 0.8429 for septic liver injury was derived from a cohort of 57 patients and has not been shown to add value beyond routine liver tests or illness-severity measures. Murine experiments provide mechanistic hypotheses for ketone-mediated organ protection, but model-specific and sometimes opposing nutritional effects limit direct translation. These metabolites are therefore best considered candidate longitudinal features for multimodal phenotyping rather than stand-alone clinical triggers. Conclusions: Biologically informed algorithmic surveillance is a promising direction, but clinical implementation requires prospective serial sampling, explicit adjustment for renal, hepatic and nutritional confounders, head-to-head comparison with routine markers, and external validation of calibration and clinical utility. Until these requirements are met, citrulline and 3-HB should support research phenotyping rather than direct treatment selection.

## 1. Introduction

Sepsis is life-threatening organ dysfunction caused by a dysregulated host response to infection, as defined by the Sepsis-3 consensus. Despite substantial investment in diagnosis and treatment, sepsis remains a major cause of morbidity and mortality [1]. Historical analyses have documented declining hospital mortality over time [2], yet survivors continue to experience important long-term complications, including increased cardiovascular risk [3]. The Global Burden of Disease analysis estimated 48.9 million sepsis cases and 11.0 million sepsis-related deaths worldwide in 2017 [4]. The timing of intervention is critical, with each hour of delayed antibiotic administration associated with a 4% to 7% increase in adult mortality risk [5] and around 7.4% in pediatric patients [6]. Protocol-driven care remains essential, but it may be complemented by predictive surveillance that identifies evolving risk before overt clinical deterioration [7]. Unlike fixed thresholds, AI models can evaluate dynamic physiological trends and subtle temporal changes that may support earlier recognition of sepsis risk [8]. Interest in clinical AI is increasing, although adoption remains constrained by electronic health record integration, model transportability, governance, and clinician trust [9]. Integrating machine learning into the intensive care unit (ICU) may support earlier risk stratification, although evidence for individualized treatment selection remains limited. While modern ICU models heavily prioritize early warning systems, true precision medicine requires more than just prediction; it demands biological context. This review explores how combining systems bioengineering, artificial intelligence, and plasma biomarkers can map the complex metabolic phenotypes of septic shock. By examining mitochondrial dysfunction alongside citrulline and 3-HB, we critically evaluate whether dynamic metabolic signatures could support biologically informed phenotyping and clarify why current evidence does not yet justify biomarker-directed interventions.

### Review Objectives & Scope

**Figure 1:**
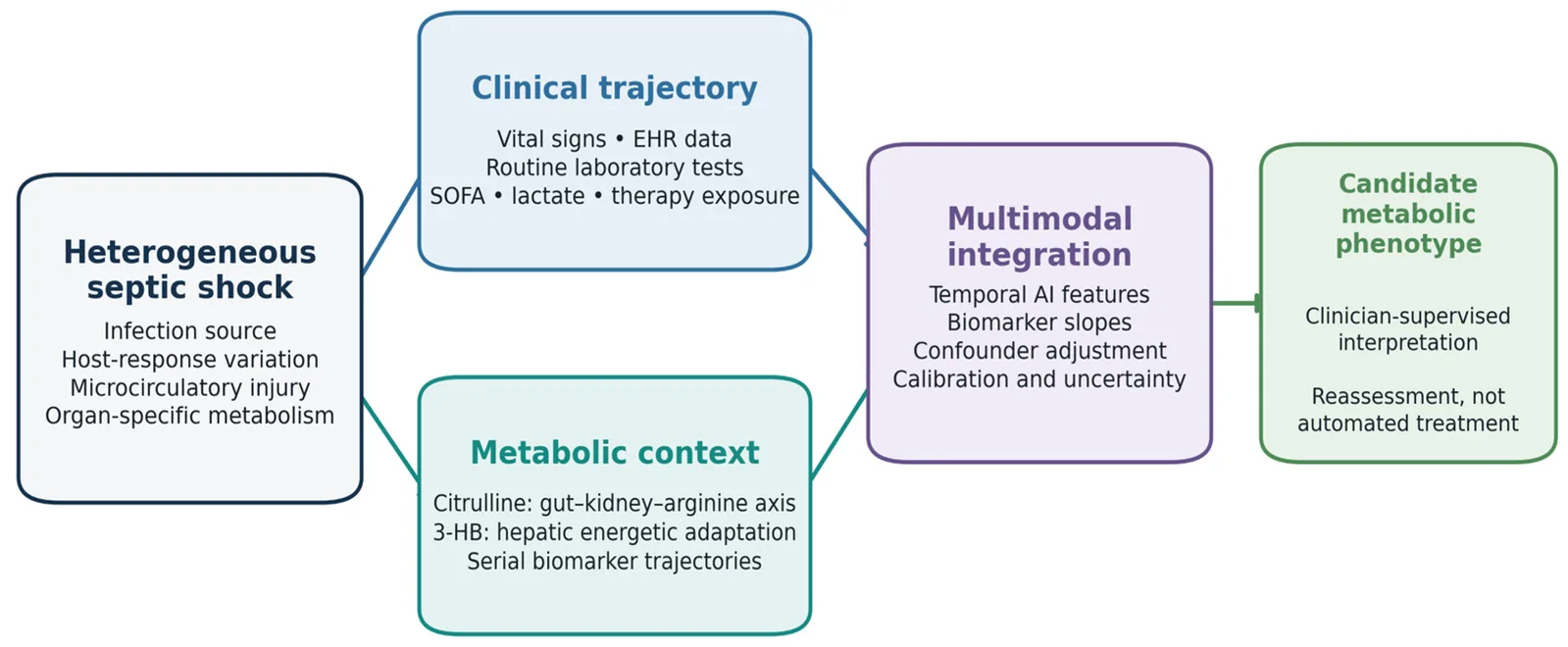
From septic-shock heterogeneity to biologically informed phenotypes.

This review examines the translational gap between algorithmic risk prediction and real-time bedside biology in sepsis management. Its primary objective is to evaluate the performance and limitations of modern machine-learning architectures compared with legacy scoring systems for early clinical detection. Beyond prediction, we map the bioenergetic disruption of septic shock through candidate metabolic phenotypes. We evaluate citrulline as a marker of enterocyte function and the gut–kidney–arginine axis, and 3-HB as a context-dependent marker of ketogenesis and hepatic metabolic reserve. We then consider how these signals might be incorporated into explainable, multimodal models after prospective validation, rather than assuming that either biomarker is currently actionable at the bedside (Figure 1).

## 2. Search Strategy and Methodology

We conducted a structured narrative review of literature indexed in PubMed/MEDLINE, Embase, Web of Science, and IEEE Xplore through June 2026. The search combined controlled vocabulary, where available, with free-text terms from three domains. Computational terms included ‘artificial intelligence,’ ‘machine learning,’ ‘explainable AI,’ ‘natural language processing,’ ‘federated learning,’ ‘early warning,’ and ‘clinical decision support.’ Biological terms included ‘metabolomics,’ ‘citrulline,’ ‘β-hydroxybutyrate,’ ‘mitochondrial dysfunction,’ and ‘metabolic phenotype.’ Translational terms included ‘sepsis,’ ‘septic shock,’ ‘multi-omics,’ ‘sepsis endotypes,’ ‘systems bioengineering,’ and ‘digital twins.’ Terms within domains were combined with OR, and the domains were linked with AND. Reference lists of relevant reviews and primary studies were examined to identify additional publications.

The review prioritized peer-reviewed human cohorts, clinical trials, prospective implementation studies, and external validations reporting clinically interpretable outcomes, discrimination, calibration, biomarker kinetics, or metabolomic phenotyping. Mechanistic animal studies were retained when they provided biological context not available from human studies. Case reports, non-peer-reviewed preprints, and in vitro studies without a clear translational link were not emphasized. Because populations, outcome definitions, sampling schedules, and validation designs were heterogeneous, no quantitative synthesis was attempted. AUROCs are therefore interpreted within their original clinical task and are not ranked across unrelated populations or outcomes.

## 3. Septic Shock Heterogeneity and Limitations of Current Assessment

Conventional sepsis assessment combines clinical examination, laboratory results, organ-dysfunction scores, and repeated reassessment. Early-warning tools such as MEWS, systemic inflammatory response syndrome (SIRS), and SOFA have shown only moderate discrimination in many cohorts; however, the commonly reported AUROCs of 0.61, 0.66, and 0.72, respectively, originate from different study populations and endpoints and should not be treated as a single head-to-head comparison. A review of 36 studies evaluating 98 AI models reported a pooled AUROC of 0.87 (95% CI, 0.86–0.88) [10]. This summary suggests promising discrimination but does not establish universal superiority because model definitions, prediction horizons, prevalence, and validation methods varied substantially. AI can nevertheless integrate longitudinal clinical information and identify multivariable patterns that fixed thresholds may not capture [6]. The Targeted Real-time Early Warning System (TREWS) illustrates both the opportunities and implementation constraints of this approach. In a multicenter prospective study, timely clinician evaluation of an alert was associated with improved outcomes, although the observational design cannot establish that the alert itself caused the mortality difference [11]. Positive predictive value, calibration, alert frequency, and workflow integration remain essential because even a model with a high AUROC can generate substantial alert burden when applied to a low-prevalence population. Successful implementation also depends on clinician and patient understanding, appropriate training, and clear accountability for final decisions [12]. AI should therefore be viewed as a cognitive adjunct to clinical judgment rather than an autonomous substitute for bedside assessment.

## 4. Artificial Intelligence as a Solution for Early Detection and Phenotyping

### 4.1. AI-Based Prediction and Early Detection

Primary prevention: From admission onward, AI models may identify trajectories associated with subsequent deterioration, particularly after major surgery or in other high-risk settings. An alert may reasonably prompt focused reassessment, closer observation, review of infection risk, and optimization of modifiable factors. It should not automatically trigger antimicrobials or invasive procedures, because false-positive predictions may lead to unnecessary testing or treatment [13].

Secondary prevention: Once infection or organ dysfunction is suspected, decision-support systems may prompt consideration of lactate measurement, complete blood count, cultures, source evaluation, and escalation to an experienced clinician. They may also support closer surveillance for acute kidney injury, respiratory failure, coagulopathy, or sepsis-associated liver injury. These prompts should remain conditional on the clinical context; fluid resuscitation, antimicrobial treatment, and vasopressor initiation require individualized evaluation rather than automatic execution of a predicted risk score [13].

Support vector machine (SVM) classifier: A supervised SVM model developed to distinguish bacterial infection in patients assessed during the COVID-19 pandemic achieved an overall AUROC of 0.84, with sensitivity ranging from 44% to 89% and specificity from 63% to 93% across evaluated settings [14]. C-reactive protein and creatinine were among the measured parameters. Because this investigation addressed bacterial infection in a specific COVID-19 context rather than septic-shock prediction, its performance cannot be compared directly with general sepsis early-warning systems. It nonetheless illustrates how routinely available blood variables may be combined within a nonlinear diagnostic model.

Advanced prediction machine-learning algorithms: Reported discrimination generally changes with the prediction horizon. One review reported AUROCs of 0.88 at sepsis onset, 0.84 at 24 h, and 0.83 at 48 h before onset [13]. These values indicate decreasing information at longer lead times and should be interpreted in the context of the original cohorts. Düsing et al. evaluated an ensemble model using latent representations and reported an AUROC of 0.92. Within the same 10-fold cross-validation framework, the model was compared with random forest plus long short-term memory (AUROC 0.86) and gradient boosting plus random forest (AUROC 0.88), with paired tests across folds yielding *p* < 0.05 [15]. Because the comparison was internal to the study, the results support model development but do not establish superiority across institutions, case definitions, or independent prospective populations.

Modern machine-learning architectures should be assessed not only by derivation performance but also by calibration, external transportability, and clinical utility. The Sepsis ImmunoScore 1.0 is an FDA-authorized AI-based software tool for sepsis risk assessment that uses 22 clinical parameters, including vital signs and laboratory variables. It reported an AUROC of 0.85 (95% CI, 0.83–0.87) in the derivation cohort, 0.80 in internal validation, and 0.81 in external validation [16]. These estimates support reproducible discrimination but do not independently demonstrate faster treatment or improved survival. In a separate study of 3204 emergency-department visits, machine-learning models using routine laboratory and administrative data predicted hospital admission with AUROCs of 0.734–0.774 and F-measures of 0.679–0.708 [17]. That study addressed disposition rather than sepsis diagnosis; its values are therefore informative within their own endpoint but are not a comparator for the Sepsis ImmunoScore. Together, these reports show why population, target outcome, prevalence, and validation design must accompany every performance estimate.

### 4.2. Multimodal Data Integration and Phenotyping

Clinical records contain both structured elements, such as vital signs, laboratory results, medications, and procedures, and unstructured elements, including progress notes, consultation reports, and imaging narratives. The proportion of unstructured information varies between institutions and cannot be represented by a universal percentage. Natural language processing (NLP) may convert parts of this information into analyzable temporal and contextual features, although portability depends on documentation practices, coding systems, missingness, and local electronic health record (EHR) configuration [18].

AI methods can combine structured EHR variables with NLP-derived features from clinical text. Such integration may identify risk signals that are absent from isolated laboratory thresholds and may support prediction at longer lead times [19]. However, narrative documentation is affected by clinician workflow, specialty vocabulary, copy-forward practices, and the timing of note entry. NLP models therefore require local validation, explicit handling of missing or delayed documentation, and testing for concept drift before their outputs are used in clinical decision support [20].

The Sepsis Early Risk Assessment (SERA) algorithm illustrates multimodal integration by combining structured data—vital signs, laboratory results, and treatments—with features extracted from unstructured notes, consultations, and pharmacy records [21]. In the same study, the diagnostic model achieved an AUROC of 0.94, sensitivity of 0.89, specificity of 0.85, and positive predictive value of 0.85 at clinical presentation. For early prediction, the AUROC was 0.87 at 48 h, 0.90 at 24 h, and 0.94 at 12 h before onset; at the 12 h horizon, sensitivity and specificity were both 0.87 [21]. Because these temporal estimates were obtained within a shared study framework, their comparison is more interpretable than numerical comparisons with models developed for different populations or endpoints. External prospective evaluation is still required to determine whether the added text-derived information improves calibration, workflow, and patient outcomes.

### 4.3. Algorithmic Endotypes and Precision Sepsis

Algorithmic endotyping seeks to divide the broad clinical syndrome of sepsis into biologically and clinically coherent subgroups. Proposed categories include a non-pulmonary sepsis phenotype (NPS), an inflammatory phenotype (INF), and an immunosuppressed, hypo-inflammatory, or dysregulated phenotype (IHD) [22]. Transcriptomic studies have likewise identified sepsis response signatures associated with different immune programs and outcomes [23]. These classifications may eventually help match patients to immunomodulatory or metabolic interventions, but endotypes depend on sampling time, assay platform, case mix, and analytic method. Their labels should not be treated as interchangeable across studies until reproducibility and treatment relevance are established.

To facilitate clinical translation, one random-forest classifier used seven routinely available clinical parameters, including alanine aminotransferase, aspartate aminotransferase, base excess, international normalized ratio, activated partial thromboplastin time, and arterial blood pressure. The study reported an AUROC of 0.95 ± 0.016, precision of 0.813 ± 0.050, and recall of 0.839 ± 0.043 on unseen test data [24]. The authors also reported a training-to-test discrepancy greater than 0.1, attributed partly to finite sample size. This apparent overfitting is important when interpreting the high test estimate. The model provides a practical hypothesis for phenotypic stratification, but independent temporal and multicenter validation is required before the phenotype assignments can be used to select treatment.

### 4.4. Clinical Implementation and Outcomes

Translating algorithmic predictions into outcomes requires more than high retrospective discrimination. Automated alerting systems may improve recognition and process measures, but effects vary with alert prevalence, clinician response, and the surrounding sepsis program [25]. In a prospective study conducted across five hospitals, 6877 patients met sepsis criteria. Confirmation of a TREWS alert within three hours was associated with an adjusted absolute mortality reduction of 3.3 percentage points (95% CI, 1.7–5.1) and a relative reduction of 18.7% (95% CI, 9.4–27.0%) [11]. The association is clinically relevant, although residual confounding remains possible because clinician response was not randomized. By contrast, external validation of a widely implemented proprietary sepsis model in 38,455 hospitalizations reported an AUROC of 0.63, sensitivity of 33%, specificity of 83%, and positive predictive value of 12% [26]. The two studies evaluated different systems and designs; together they emphasize that local external validation and prospective workflow assessment are essential.

Reports of reduced mortality, length of stay, or readmission after implementation should be interpreted with attention to baseline trends, concurrent quality-improvement measures, and institutional case mix. Observational before-and-after findings may reflect a contribution from the model, but they cannot isolate the algorithm from changes in clinical workflow. Organ-specific prediction provides another illustration of endpoint dependence. Lei et al. developed a model for sepsis-associated liver injury (SALI) in which total bilirubin, lactate, prothrombin time, and mechanical ventilation were influential predictors [27]. Its AUROC decreased from 0.838 in internal testing to 0.721 during external validation, demonstrating the common attenuation that occurs when models are transported to another hospital system. These SALI estimates cannot be compared directly with AUROCs for sepsis onset, mortality, or metabolomic phenotypes because the clinical target differs. Clinician perception studies also show that alert timing, interpretability, and perceived relevance influence whether a prediction changes management [28]. Reviews of the field consistently identify heterogeneous outcome definitions, limited calibration reporting, and insufficient external or prospective validation as barriers to implementation [10,13]. Future studies should combine rigorous model evaluation with prospective comparison of AI-augmented care and usual care, while reporting workflow effects, unintended consequences, and patient-centered outcomes.

## 5. Metabolic Biomarkers as Biological Context

### 5.1. Metabolic Dysfunction in Septic Shock

Septic shock involves an interacting disturbance of inflammation, perfusion, endothelial regulation, substrate use, and cellular bioenergetics. Mitochondrial dysfunction may impair oxidative phosphorylation even when global oxygen delivery appears adequate, while microcirculatory heterogeneity can create regional oxygen and nutrient deficits. Accumulation of tricarboxylic acid cycle intermediates, altered fatty-acid oxidation, and changes in the NAD+/NADH balance have therefore been proposed as components of an evolving metabolic phenotype rather than as a single universal mechanism [29].

Serum lactate remains an important bedside marker, but it is influenced by production, hepatic clearance, adrenergic stimulation, and mitochondrial metabolism and is not specific for tissue hypoxia. Targeted and untargeted metabolomics can identify complementary pathway-level changes, including altered fatty-acid metabolism and tricarboxylic acid cycle intermediates. One integrated clinico-metabolomic model reported an AUROC of 0.82 for mortality in its study population [30]. This endpoint-specific estimate should be interpreted within that cohort and should not be ranked against SOFA, citrulline studies, SALI models, or early-warning algorithms developed for different outcomes.

### 5.2. Citrulline as a Marker of Intestinal, Endothelial and Nitric Oxide-Related Dysfunction

Citrulline occupies a defined gut–kidney metabolic axis. In the postabsorptive state, small-intestinal enterocytes synthesize most circulating citrulline from glutamine and proline; citrulline largely bypasses hepatic uptake and is converted by the proximal kidney into arginine [31,32,33]. Septic shock can disrupt this flux through splanchnic hypoperfusion, impaired substrate delivery, inflammatory suppression of enterocyte metabolism and epithelial injury. Stable-isotope tracing provides direct human evidence: in 10 patients with septic shock, whole-body citrulline production was 4.5 ± 2.1 μmol·kg^−1^·h^−1^, compared with 10.1 ± 2.9 in critically ill non-septic controls and 13.7 ± 4.1 in healthy controls; de novo arginine and nitric oxide production were also reduced [34]. Thus, low plasma citrulline in sepsis reflects reduced metabolic production and disruption of the intestine–kidney–arginine pathway, rather than merely a nonspecific change in amino-acid concentration.

Mechanistically, loss of enterocyte-derived citrulline constrains renal and local arginine regeneration. Arginine scarcity may contribute to endothelial dysfunction by limiting nitric oxide synthase substrate availability; under substrate-limited conditions, nitric oxide synthase uncoupling and oxidant formation have been proposed, although this causal sequence has not been demonstrated directly in septic patients [35,36]. In 135 patients with severe sepsis, median plasma citrulline was 9.2 μmol/L (interquartile range 5.2–14.4); concentrations were lower in patients with acute respiratory distress syndrome than in those without it (6.0 versus 10.1 μmol/L), and acute respiratory distress syndrome occurred in 50% of patients in the lowest citrulline quartile compared with 15% in the highest quartile [37]. The association persisted after adjustment for illness severity. These findings link hypocitrullinemia to organ injury, but do not establish that citrulline supplementation improves outcomes.

**Figure 2:**
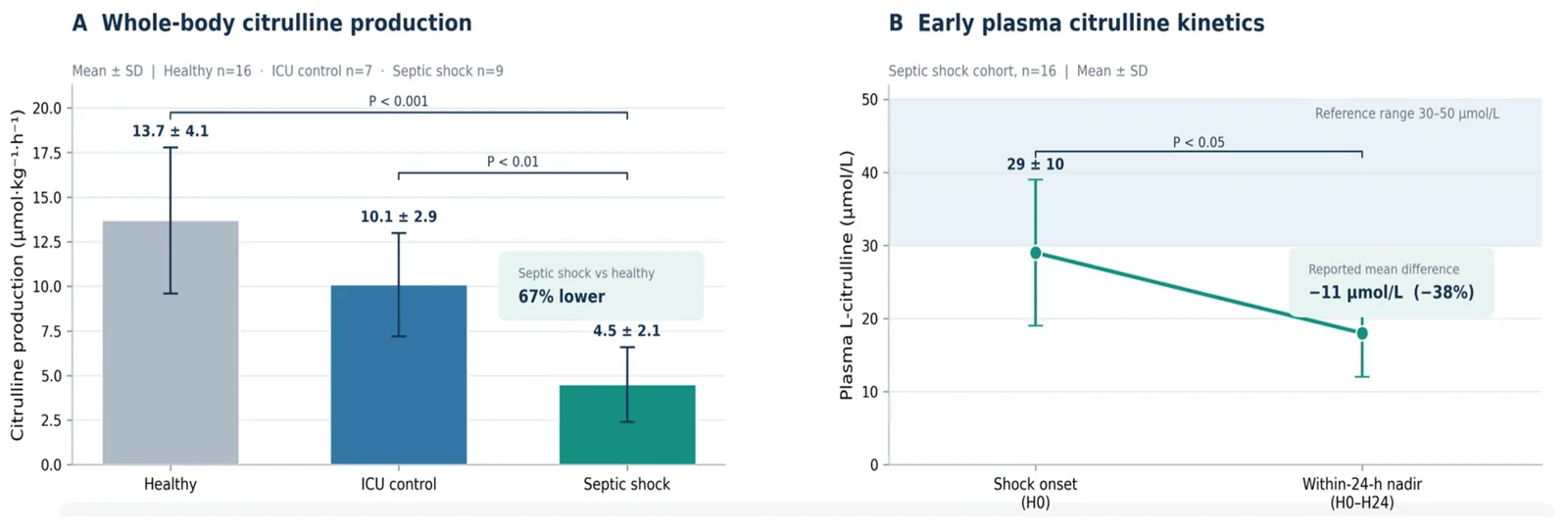
Human Evidence for altered Citrulline metabolism in septic shock.

The limited kinetic data support serial rather than single-point interpretation. In a 16-patient septic shock pilot study, mean plasma citrulline fell from 29 ± 10 μmol/L at shock onset to a nadir of 18 ± 6 μmol/L during the first 24 h). The nadir was lower in patients with digestive bacterial translocation and citrulline was inversely correlated with C-reactive protein, but concentrations did not distinguish survivors from non-survivors [38]. In another prospective study of 28 patients with sepsis and 30 with septic shock, citrulline was lower in the shock group throughout serial sampling on days 1, 3, 5, 7 and 10; it was also lower with acute gastrointestinal injury grade III on days 7 and 10, yet did not differ between survivors and non-survivors [39]. The direction supported by septic shock data is therefore low or persistently low citrulline, not elevated citrulline (Figure 2). 

No clinical decision cutoff has been validated specifically for septic shock. Postabsorptive concentrations of approximately 30–50 μmol/L are generally considered normal, whereas the frequently cited 20 μmol/L threshold was derived mainly from chronic intestinal failure and enteropathy rather than acute sepsis [31,40]. Published sepsis studies report cohort-specific ranges, trajectories or quartiles rather than a reproducible treatment threshold. Renal dysfunction can increase plasma citrulline by reducing renal uptake and conversion to arginine, potentially masking diminished intestinal production; nutritional exposure, pre-existing intestinal resection or disease, mucositis and assay timing introduce further variability. Findings from chemotherapy-related mucositis or febrile neutropenia should therefore not be extrapolated to claim that elevated citrulline predicts a complicated septic shock course [41,42].

### 5.3. β-Hydroxybutyrate as a Marker of Energetic Stress and Mitochondrial Adaptation

β-Hydroxybutyrate (3-HB) is produced predominantly by hepatic ketogenesis during low insulin availability, fasting, or increased fatty-acid delivery. It serves both as an oxidative fuel and as a signaling metabolite, but its circulating concentration reflects the balance among hepatic production, tissue uptake, and ketolysis. In septic shock, a low concentration may indicate impaired hepatic ketogenesis or altered gut–liver metabolic signaling, whereas a high concentration may reflect adaptive fasting, diabetes, exogenous lipid delivery, or impaired peripheral utilization. Consequently, neither direction is intrinsically protective or harmful without metabolic and nutritional context.

Human evidence remains preliminary and organ-specific. In the study by Hu et al., serum 3-HB was lower in 17 septic patients with liver injury than in 40 septic patients without liver injury and correlated inversely with alanine aminotransferase, aspartate aminotransferase, total bilirubin and ICU length of stay [43]. The reported AUROC of 0.8429 describes discrimination of septic liver injury within this 57-patient cohort; it is not an AUROC for sepsis detection, septic shock mortality or treatment response. The study did not provide a validated clinical cutoff, external validation, calibration or decision-curve analysis, and did not establish incremental value over routinely available alanine aminotransferase, aspartate aminotransferase, bilirubin, international normalized ratio, lactate or SOFA. Its AUROC therefore should not be directly compared with biomarkers or scores developed for different clinical endpoints.

Preclinical evidence offers biological plausibility but not direct clinical proof. In murine models, time-restricted feeding increased Lactobacillus murinus and 3-HB, while genetic disruption of hepatic Hmgcs2 or downstream Lpin1 weakened the protective pathway; complementary cell and mouse experiments implicated PI3K/AKT/mTOR/LPIN1 signaling and reduced hepatocyte ferroptosis [43]. Exogenous 3-HB has also attenuated neuroinflammation and metabolic disruption in murine sepsis-associated encephalopathy [44]. However, ketogenic nutrition combined with PPARα activation worsened metabolic failure and muscle weakness in another septic mouse model [45]. Differences in dose, route, timing, organ endpoint, nutrition and sepsis model probably account for part of this divergence. These interventions cannot be translated into a recommendation for ketogenic feeding or 3-HB supplementation in human septic shock.

Clinical interpretation also requires explicit control of kinetics and confounding. A standardized sampling window or sepsis-specific 3-HB cutoff has not been established. Fasting duration, caloric and lipid delivery, insulin and catecholamine exposure, diabetes or ketoacidosis, sodium–glucose cotransporter-2 inhibitor use, hepatic function, renal clearance and renal replacement therapy can all change circulating ketones. Until prospective studies define reproducible trajectories and demonstrate added value over routine liver and metabolic tests, 3-HB should be treated as an investigational marker of a possible hepatometabolic phenotype rather than a stand-alone diagnostic or therapeutic target.

### 5.4. Practical Integration into Clinical Decision-Making

The near-term role of both metabolites is adjunctive stratification, not autonomous decision-making. A candidate prospective protocol would obtain plasma citrulline and 3-HB at ICU admission, preferably before major nutritional changes, and repeat sampling at approximately 24 h; later citrulline measurements could characterize gastrointestinal recovery or persistent dysfunction. A falling or persistently low citrulline concentration, when concordant with the acute gastrointestinal injury score, feeding intolerance, I-FABP, lactate and vasopressor requirements, could prompt closer evaluation of gastrointestinal perfusion and integrity. Similarly, low 3-HB together with rising alanine aminotransferase, aspartate aminotransferase, bilirubin or international normalized ratio could support assignment to a hepatometabolic injury phenotype. Neither pattern should independently diagnose intestinal ischemia or liver injury, trigger supplementation, or replace established clinical assessment.

For systems-bioengineering and AI models, absolute biomarker values should be accompanied by their slopes, sampling time and key modifiers, including renal function and renal replacement therapy for citrulline and feeding, insulin, diabetes and hepatic function for 3-HB. Prospective validation should predefine the intended endpoint, assay and sampling window; compare added discrimination with SOFA, lactate and organ-specific laboratory tests; and report calibration, external validation and decision-curve utility. This framework preserves the biological information carried by the metabolites while preventing premature conversion of exploratory associations into bedside treatment rules.

## 6. Integration Through Systems Bioengineering

### 6.1. Plasma Biomarker Integration for Metabolic Phenotyping

A systems-bioengineering framework does not interpret biomarkers as isolated laboratory values, but as dynamic nodes within an interconnected network linking mitochondrial function, endothelial perfusion, immune activation, and organ dysfunction. Citrulline and 3-HB provide targeted information about enterocyte metabolism and hepatometabolic adaptation, respectively, but biological heterogeneity makes a single-biomarker strategy insufficient for reliable prognostication [46]. Their most plausible role is within a multiplexed plasma profile that also includes lactate, inflammatory markers, renal and hepatic indices, nutrition, and clinical trajectories [47]. Such integration may define coherent metabolic subphenotypes without assuming that either metabolite is a stand-alone treatment trigger.

**Figure 3:**
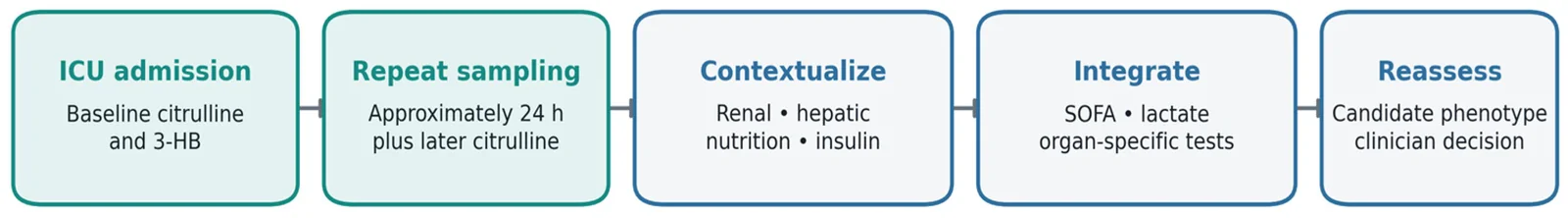
Proposed Prospective Workflow for Integrating Citrulline and 3-Hydroxybutyrate into Septic Shock Phenotyping.

This approach depends on high-throughput platforms, particularly liquid chromatography–mass spectrometry (LC-MS) and quantitative nuclear magnetic resonance (NMR) spectroscopy, which can quantify broad panels of circulating metabolites. Foundational studies linked abnormalities in fatty-acid oxidation, gluconeogenesis, and the tricarboxylic acid cycle with mortality [48]. Plasma metabolome profiling has also identified early sepsis clusters characterized partly by lipid alterations and metabolic-network dysfunction [49]. These findings support stratification by biochemical mechanism rather than organ-dysfunction score alone, although assay harmonization, batch control, and external validation remain necessary before routine clinical application (Figure 3) [50].

**Figure 4:**
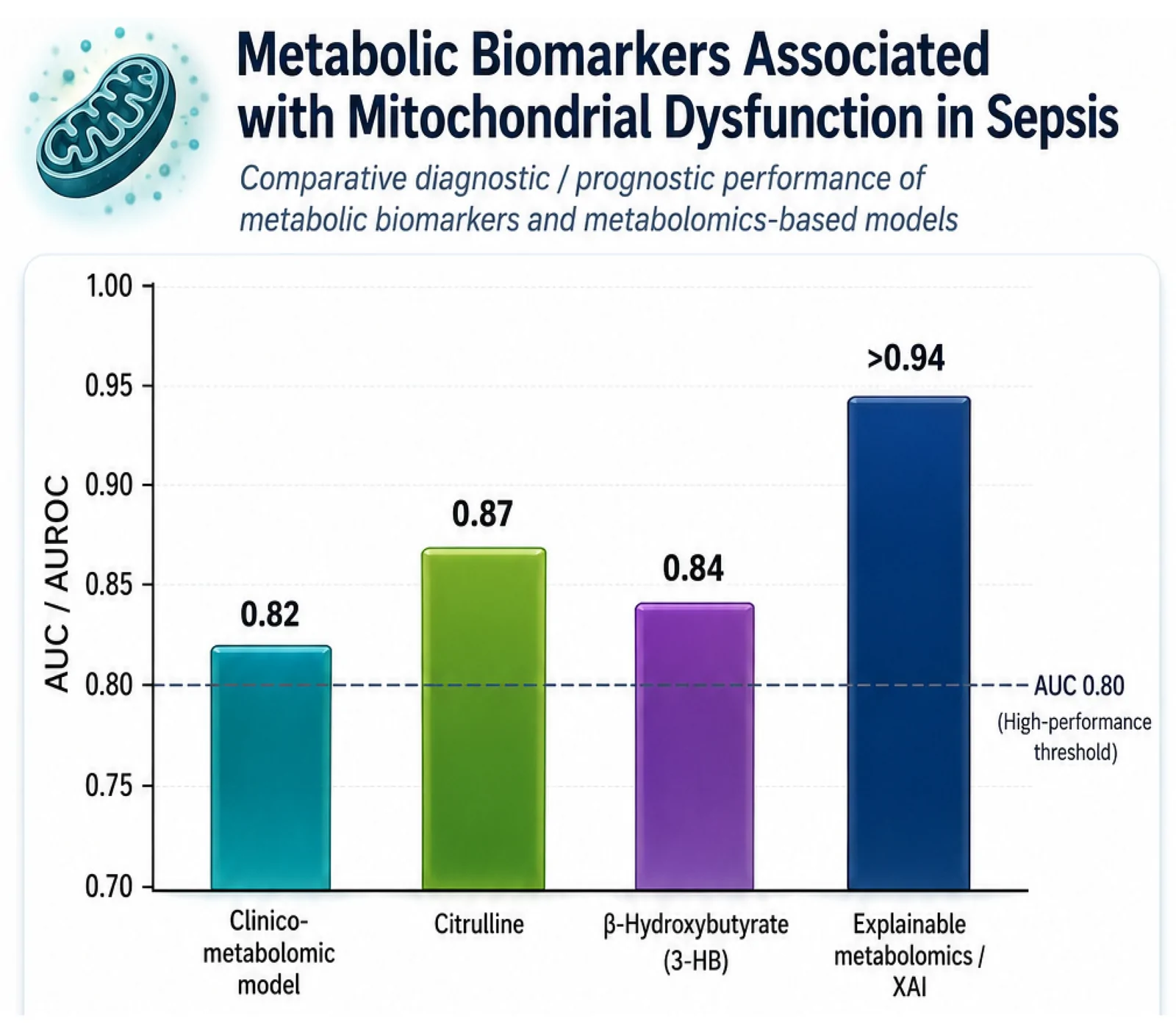
Biomarkers versus Explainable metabolomics.

Multi-omic studies have described biological signatures in sepsis-associated acute kidney injury (SA-AKI) [51] and in immunosuppressive endotypes characterized by altered endothelial and immune metabolism [52]. Explainable AI applied to platelet metabolite data identified interactions involving carnitine, glutamate, and myo-inositol and reported a diagnostic AUROC above 0.94 in the population studied [53]. Untargeted LC-MS analysis in patients with polytrauma-associated sepsis identified candidate clusters that included succinic acid semialdehyde and uridine [54], while machine learning combined with NMR-based metabolomics showed prognostic potential in severe septic shock [55]. These studies differ in sample type, population, endpoint, and validation design; their AUROCs should therefore not be ranked against early-warning algorithms or organ-injury biomarkers. Their main value is to define reproducible multimodal features that can be evaluated prospectively within systems-bioengineering models (Figure 4). 

### 6.2. Systems Bioengineering Approach: From Isolated Biomarkers to Metabolic Signatures

The integration of multi-omics and machine learning has generated candidate biomarkers such as mitogen-activated protein kinase 14 (MAPK14) and KRAS in neonatal sepsis, with reported external-validation AUROCs above 0.79 [56]. The analysis linked these candidates with ferroptosis- and cuproptosis-related pathways, but reliance on public datasets and a neonatal population limits generalization to adult septic shock. A separate multi-cohort transcriptomic study identified PPARG as a candidate diagnostic biomarker and reported an external-validation AUROC of 0.95 [57]. Experimental analyses implicated CD14/NF-κB signaling, providing a mechanistic hypothesis rather than proof of a clinically actionable therapeutic target. The two AUROC estimates address different populations and analytic tasks and should not be compared directly [27].

Computational phenotyping can move beyond static organ-dysfunction scores by identifying hyperinflammatory, coagulopathic, hepatobiliary, and multi-organ-failure patterns. The Alpha, Beta, Gamma, and Delta phenotypes described by Seymour et al. were associated with distinct clinical characteristics, outcomes, and possible treatment interactions [58]. These groups should not be considered fixed identities. In an externally validated dynamic-phenotyping study, 48.3% of patients changed phenotype within six hours [59]. Phenotypic mobility may be clinically informative, but it complicates treatment assignment and demonstrates why sampling time and repeated classification must be specified. Prospective studies are needed to determine whether following phenotype transitions improves treatment selection compared with conventional repeated clinical assessment.

Potential treatment-effect heterogeneity has been explored in secondary and retrospective analyses. In the VANISH randomized trial, the SRS2 transcriptomic endotype showed higher mortality with hydrocortisone than with placebo (44% vs. 10%), whereas this pattern was not observed in SRS1 [60]. Because this was a secondary subgroup analysis, it remains hypothesis-generating and should not be used alone to withhold or prescribe corticosteroids. Other studies have associated hourly norepinephrine-equivalent trajectories over 72 h with phenotype-specific risk, including a U-shaped dose–mortality relationship within a severe multi-organ-failure subgroup [61]. Unsupervised analyses have also examined whether recombinant human thrombomodulin may be more beneficial in patients with profound coagulopathy and advanced organ dysfunction than in less severe phenotypes [62,63]. These observations illustrate the promise of treatment-response phenotyping, but prospective enrichment trials are required before any signature guides routine therapy.

## 7. Implementation Challenges and Future Directions

### 7.1. Explainability, Alert Fatigue and Clinician Trust

Clinical translation is influenced by alert burden, limited transparency, and uncertainty about how a prediction should change management. In a clinician-perception study linked to a sepsis early-warning implementation, 180 nurses and 107 providers evaluated 362 alerts; 30% of nurses and 9% of providers reported that an alert prompted a management change [28]. These results should not be interpreted as general rejection of AI. Rather, they show that perceived usefulness depends on timing, specificity, familiarity with the model, and integration with bedside reasoning. A technically accurate alert may still be ignored when it is repetitive, poorly timed, or disconnected from an actionable clinical pathway.

Explainable artificial intelligence (XAI) frameworks can reveal which variables contribute to an individual prediction and may help clinicians judge whether the model is responding to plausible physiology or to an artifact [64]. Feature-attribution approaches such as SHapley Additive exPlanations (SHAP) and local interpretable model-agnostic explanations (LIME) can summarize the direction and relative influence of variables such as lactate, blood pressure, renal indices, or medication exposure [64,65]. These methods may support model review and communication, but they do not establish causal relationships, correct biased training data, or guarantee clinical benefit. Explanations should therefore be presented with calibration, uncertainty, data-quality warnings, and a clear statement that the clinician retains responsibility for interpretation.

Lei et al. combined LightGBM, XGBoost, and random-forest models with SHAP summaries to clarify influential features in SALI prediction [27]. This approach may make a complex ensemble easier to interrogate, although feature importance remains model- and dataset-dependent. Excessive non-actionable alerts can still cause cognitive overload, particularly during busy shifts or when a patient differs from the development population. Human–AI alignment should therefore be evaluated as part of model performance. In the TREWS deployment study, 82% of retrospective sepsis cases were identified, clinicians evaluated 89% of alerts, and sepsis was confirmed in 38% of evaluated alerts [66]. Provider adoption was associated with treatment timing, but these process findings should not be interpreted as proof that explanation alone improves survival. Future systems should pair concise alerts with the principal contributing variables, recommended reassessment steps, escalation pathways, and continuous measurement of override rates, alert fatigue, and unintended consequences.

### 7.2. Regulation, Ethics and Data Privacy

Regulatory oversight of clinical AI increasingly emphasizes evidence of safety, performance, transparency, and post-deployment monitoring. The appropriate pathway depends on whether software merely organizes information or functions as a medical device that influences diagnosis or treatment. In the European Union and United States, developers and institutions must consider data governance, risk classification, human oversight, and the effect of model updates [67]. Regulatory authorization demonstrates that specified requirements have been addressed; it does not remove the need for local validation or continuing surveillance in the intended population.

Models trained on unrepresentative datasets may perform differently across sex, age, ethnicity, socioeconomic groups, hospitals, or patterns of comorbidity. Equity assessment should include subgroup discrimination and calibration, error analysis, missing-data patterns, and access to the clinical response triggered by an alert. Transparent documentation of development populations and continuous auditing are therefore central to responsible deployment [67,68].

Federated learning offers a decentralized approach in which institutions train a shared model without routinely moving raw patient records to a central repository. Participating sites exchange model updates rather than full clinical datasets, which may reduce some data-sharing barriers and enable exposure to more diverse populations. Mondrejevski et al. demonstrated the feasibility of deep federated learning for sepsis prediction near the first hour of a defined five-hour SIRS interval [69]. However, decentralization does not automatically remove bias, site-specific calibration differences, privacy leakage from model updates, or unequal data quality. Claims of a fixed percentage reduction in discrimination against minority groups are not supported without direct comparative evaluation. The General Data Protection Regulation and Health Insurance Portability and Accountability Act provide important legal baselines, but AI governance must also address data minimization, access control, cybersecurity, auditability, secondary use, and the possibility of re-identification [67,68]. Federated architectures are therefore a promising technical component of multi-institutional validation rather than a complete solution to privacy or generalizability.

### 7.3. Multi-Omics, Digital Twins and Future Directions

Future sepsis models may combine clinical data with genomics, epigenomics, transcriptomics, proteomics, and metabolomics to describe the evolving immune and metabolic state [70,71]. Such integration could identify subgroups relevant to antimicrobial, immunomodulatory, or supportive treatment, but current evidence does not justify replacing empiric treatment with an automatically generated molecular recommendation. Sampling time, batch effects, missing modalities, cost, and limited treatment-linked validation remain substantial barriers. Prospective studies should test whether multi-omic information improves calibration, decisions, or patient outcomes beyond well-specified clinical baselines and should report the resources required to obtain results within a useful clinical timeframe.

Genomic information provides a relatively stable layer that may characterize inherited host susceptibility, immune-response variation, and pharmacogenomic differences. Sepsis is not generally driven by a small group of somatic ‘driver mutations,’ and individual variants should not be assumed to determine outcome. Integrating genomic variation with dynamic transcriptomic and proteomic measurements may identify pathways associated with susceptibility or response, but analyses must address ancestry, population structure, multiple testing, and replication across clinical settings [72]. Falling sequencing costs improve feasibility, while data volume, interpretation, consent, and equitable representation remain important constraints.

**Figure 5:**
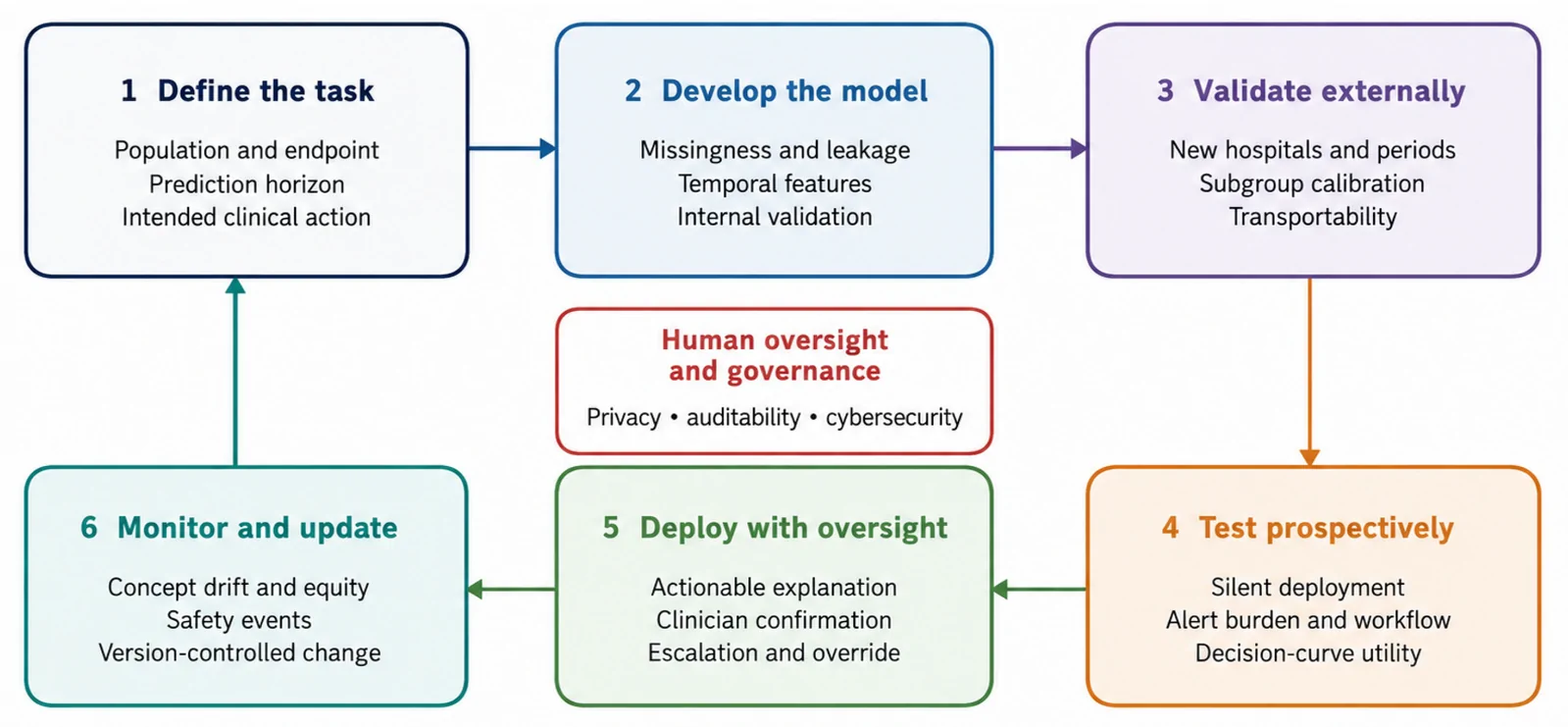
Lifecycle Framework for Responsible Clinical AI Development and Implementation in Sepsis.

Transcriptomics provides a time-sensitive readout of the host response and can identify patterns of inflammatory activation, T-cell exhaustion, or immunoparalysis that may precede or accompany clinical deterioration. AI-based pipelines can analyze gene-expression networks rather than isolated transcripts and may identify candidate regulatory nodes. PPARG is one such candidate, but current evidence does not establish this pathway as the single driver of systemic inflammation or as a validated treatment target [57,73]. Transcriptomic results are sensitive to cell composition, sampling time, treatment exposure, and technical platform. Integration with proteomic and metabolomic measurements may improve biological interpretation, provided that the combined model is externally validated (Figure 5).

#### 7.3.1. Continuous Monitoring and Wearables

Wearable sensors may extend physiological monitoring beyond the ICU and provide higher-frequency inputs for risk models. A study in low- and middle-income settings explored wearable-derived features for sepsis mortality prediction [74]. Before wider use, signal quality, missing data, battery and network limitations, patient comfort, and prospective clinical utility require evaluation. Cloud transmission also introduces privacy, latency, and interoperability requirements that vary between hospitals and community settings.

#### 7.3.2. Adaptive Models and Concept-Drift Control

Changes in patient populations, clinical practice, coding, laboratory platforms, or antimicrobial resistance may degrade model performance over time. Monitoring input distributions, calibration, and subgroup error rates can identify concept drift [20]. Model updating should occur under version control with predefined validation, change documentation, and governance rather than through unsupervised bedside retraining that could alter performance without clinical review.

#### 7.3.3. Digital Twins and Causal Inference

Patient-specific computational models could eventually combine physiological trajectories, treatment exposure, and molecular measurements to simulate plausible responses to fluids, vasopressors, antibiotics, or other interventions [75]. This concept is attractive because septic shock evolves rapidly and the effect of treatment depends on timing and baseline physiology. At present, most healthcare digital twins remain conceptual or early-stage models. Their outputs are sensitive to structural assumptions, incomplete measurements, data latency, and unmeasured confounding. Causal-inference methods may help distinguish treatment effects from associations, but valid counterfactual estimation requires explicit assumptions, positivity, adequate covariate measurement, and appropriate study designs. Multi-omic integration may enrich digital representations of the host response [70,76], yet it also increases dimensionality, batch effects, and missing-data problems. Current evidence does not justify autonomous order entry or the claim that a digital twin can ensure an optimal treatment before bedside administration. Near-term research should focus on transparent models that support clinician-supervised scenario analysis and are prospectively compared with standard decision-making.

#### 7.3.4. Precision Medicine and Post-Sepsis Recovery

Precision models could be extended beyond acute mortality to outcomes relevant to survivors, including recurrent infection, cardiovascular events, cognitive and functional impairment, readmission, nutrition, and rehabilitation needs [3]. Longitudinal EHR text, socioeconomic variables, patient-reported outcomes, and wearable data may help characterize recovery, but each prediction horizon and endpoint requires separate development and validation. Current evidence does not support claims that models can accurately simulate an individual 30-day, 90-day, or one-year recovery trajectory or reproduce the specificity of an unrelated diagnostic SVM. Post-sepsis tools should therefore support multidisciplinary follow-up, identify patients who may benefit from reassessment, and communicate uncertainty rather than prescribe a fully automated rehabilitation pathway [13,77]. Sensor miniaturization and cloud computing may improve data availability, but clinical benefit must be demonstrated independently of technical feasibility.

## 8. Conclusions

The review recognizes septic shock as a systems-level metabolic disorder in which mitochondrial dysfunction, endothelial injury, immune dysregulation and organ failure evolve together. Artificial intelligence may improve early recognition by detecting subtle clinical trajectories, but prediction becomes more interpretable when linked to measurable biology. This review therefore treats citrulline and 3-HB as candidate, not established, phenotyping biomarkers. Human evidence supports low or persistently low citrulline as a signal of disrupted enterocyte function and the gut–kidney–arginine axis, while the clinical evidence for 3-HB is limited to a small organ-specific cohort and requires careful nutritional and metabolic context. If prospectively validated, their trajectories could complement lactate and routine organ-function tests within a broader plasma signature of septic shock metabolism. Integrating citrulline, 3-HB, multiplexed metabolomics, ICU clinical data and explainable AI may eventually distinguish patients with similar sepsis presentations but different metabolic states. Before clinical implementation, these systems require standardized serial sampling, explicit adjustment for renal, hepatic and nutritional confounders, head-to-head comparison with routine markers, and external multicenter validation of calibration and clinical utility. The appropriate near-term goal is therefore clinician-supervised research phenotyping, not automated diagnosis, biomarker-triggered supplementation or treatment selection.

## Data Availability

The original contributions presented in this study are included in the article. Further inquiries can be directed to the corresponding author.

## Abbreviations

The following abbreviations are used in this manuscript:

3-HB	3-hydroxybutyrate (β-hydroxybutyrate)
AI	Artificial intelligence
ALT	Alanine aminotransferase
aPTT	Activated partial thromboplastin time
AST	Aspartate aminotransferase
ATP	Adenosine triphosphate
AUROC	Area under the receiver operating characteristic curve
CBC	Complete blood count
CD14	Cluster of differentiation 14
CI	Confidence interval
CRP	C-reactive protein
DNA	Deoxyribonucleic acid
EHR	Electronic health record
EU	European Union
FDA	Food and Drug Administration
FL	Federated learning
GB	Gradient boosting
GDPR	General Data Protection Regulation
HIPAA	Health Insurance Portability and Accountability Act
Hmgcs2	3-hydroxy-3-methylglutaryl-CoA synthase 2
ICU	Intensive care unit
I-FABP	Intestinal fatty acid-binding protein
IHD	Immunosuppressed/hypo-inflammatory/dysregulated phenotype
INF	Inflammatory phenotype
INR	International normalized ratio
IV	Intravenous
KRAS	Kirsten rat sarcoma viral oncogene homolog
LC-MS	Liquid chromatography–mass spectrometry
LIME	Local interpretable model-agnostic explanations
LOS	Length of stay
LSTM	Long short-term memory
MAPK14	Mitogen-activated protein kinase 14
MEWS	Modified Early Warning Score
ML	Machine learning
MLA	Machine learning algorithm
NAD+/NADH	Oxidized/reduced nicotinamide adenine dinucleotide
NEE	Norepinephrine equivalent
NF-κB	Nuclear factor kappa-light-chain-enhancer of activated B cells
NLP	Natural language processing
NMR	Nuclear magnetic resonance
NO	Nitric oxide
NPS	Non-pulmonary sepsis phenotype
PPARG	Peroxisome proliferator-activated receptor gamma
PPV	Positive predictive value
RCT	Randomized controlled trial
RF	Random forest
rhTM	Recombinant human thrombomodulin
SA-AKI	Sepsis-associated acute kidney injury
SALI	Sepsis-associated liver injury
SERA	Sepsis Early Risk Assessment
SHAP	SHapley Additive exPlanations
SIRS	Systemic inflammatory response syndrome
SOFA	Sequential Organ Failure Assessment
SRS1	Sepsis Response Signature 1
SRS2	Sepsis Response Signature 2
SVM	Support vector machine
TCA	Tricarboxylic acid
TREWS	Targeted Real-time Early Warning System
VANISH	Vasopressin versus norepinephrine as initial therapy in septic shock
XAI	Explainable artificial intelligence
XGBoost	Extreme Gradient Boosting
